# Supplementary material for: Evaluating the accuracy of Salmonella Typhi Hemolysin E and lipopolysaccharide IgA to discriminate enteric fever from other febrile illnesses in South Asia
Source: medRxiv. 2025 Jun 22:2025.06.20.25329792. Preprint. [Version 1] doi: 10.1101/2025.06.20.25329792 (PMC12204246; doi:10.1101/2025.06.20.25329792)
Supplement: Supplement 2 [file media-2.pdf]

Appendix Table 1. Diagnostic criteria and methods for alternative etiology febrile controls*

Etiology	Diagnostic inclusion criteria
Dengue	Positive IgM serology OR NS1 antigen positive
Malaria	RDT positive OR blood smear positive
Scrub Typhus	Positive IgM serology
Other Bacteremia	Positive blood culture† excluding likely contaminants (e.g. coagulase negative *Staph spp*s, *Micrococcus*, *Bacillus sp*p, etc)
COVID-19	Positive PCR or rapid antigen test

* NS1= non-structural protein 1, PCR = polymerase chain reaction, RDT= rapid diagnostic test
†Blood culture was performed from whole blood using automated culture systems (BACTEC, Becton Dickinson, Franklin Lakes, NJ, USA; BacTAlert 3D, BioMérieux, Marcy-l'Étoile, France
