# Supplementary material for: Evaluating the accuracy of Salmonella Typhi Hemolysin E and lipopolysaccharide IgA to discriminate enteric fever from other febrile illnesses in South Asia
Source: medRxiv. 2025 Jun 22:2025.06.20.25329792. Preprint. [Version 1] doi: 10.1101/2025.06.20.25329792 (PMC12204246; doi:10.1101/2025.06.20.25329792)
Supplement: Supplement 4 [file media-4.pdf]

Appendix Table 3. Sensitivity analysis of the inclusion criteria for number of days of fever at clinical presentation

				HlyE IgA*		LPS IgA		HlyE + LPS IgA*	
**Site**	**Days of Fever**	**Case (N)**	**Control (N)**	**AUC**	**p value**	**AUC**	**p value**	**AUC**	**p value**
**All**	≤3	200	123	0.82 (0.78, 0.87)	ref	0.92 (0.89, 0.95)	ref	0.93 (0.90, 0.96)	ref
	4-5	223	69	0.89 (0.86, 0.93)	0.02	0.94 (0.92, 0.97)	0.22	0.96 (0.94, 0.98)	0.06
	6-14	227	71	0.89 (0.84, 0.93)	0.05	0.88 (0.84, 0.93)	0.18	0.91 (0.87, 0.95)	0.50
**Bangladesh**	≤3	120	47	0.87 (0.81, 0.93)	ref	0.94 (0.89, 0.98)	ref	0.94 (0.90, 0.99)	ref
	4-5	146	19	0.96 (0.93, 1.00)	0.01	0.96 (0.93, 0.99)	0.43	0.98 (0.97, 1.00)	0.09
	6-14	145	13	0.94 (0.89, 0.99)	0.07	0.90 (0.84, 0.96)	0.31	0.94 (0.90, 0.98)	0.93
**Nepal**	≤3	70	56	0.71 (0.62, 0.80)	ref	0.91 (0.85, 0.97)	ref	0.89 (0.83, 0.95)	ref
	4-5	48	19	0.74 (0.62, 0.85)	0.70	0.92 (0.84, 0.99)	0.91	0.92 (0.85, 0.99)	0.56
	6-14	37	27	0.81 (0.70, 0.92)	0.17	0.90 (0.81, 0.98)	0.81	0.89 (0.79, 0.98)	0.94
**Pakistan**	≤3	10	20	0.98 (0.95, 1.00)	ref	0.98 (0.94, 1.00)	ref	1.00 (1.00, 1.00)	ref
	4-5	29	31	0.90 (0.82, 0.99)	0.08	0.95 (0.89, 1.00)	0.51	0.96 (0.89, 1.00)	0.24
	6-14	45	31	0.90 (0.82, 0.97)	0.89	0.90 (0.83, 0.97)	0.29	0.92 (0.86, 0.99)	0.44

* HlyE = Hemolysin E, LPS = Lipopolysaccharide, AUC = Area Under the Curve
