# Supplementary material for: Evaluating the accuracy of Salmonella Typhi Hemolysin E and lipopolysaccharide IgA to discriminate enteric fever from other febrile illnesses in South Asia
Source: medRxiv. 2025 Jun 22:2025.06.20.25329792. Preprint. [Version 1] doi: 10.1101/2025.06.20.25329792 (PMC12204246; doi:10.1101/2025.06.20.25329792)
Supplement: Supplement 5 [file media-5.pdf]

Appendix Table 4. Modeled longitudinal kinetic parameter estimates

Parameter	Description	Units	HlyE IgA Median (Q1, Q3)	LPS IgA* Median, (Q1, Q3)
**Decay Rate (alpha)**	The rate at which antibody concentrations decline during the waning phase after the peak response.<br>-Smaller values indicate slower decay	Days	0.00031<br>(0.00015, 0.00062)	0.00035<br>(0.00011, 0.0010)
**Shape Factor (r)**	Describes the nonlinearity of antibody decay:<br>- When r>1r > 1, decay starts rapidly and slows over time, deviating from exponential decay<br>- Higher r values indicate faster early decay, transitioning to slower decay later	Dimensionless	2.07<br>(1.80, 2.40)	2.35<br>(2.01, 2.81)
**Time to Peak (t1)**	Represents the time taken to reach the maximum antibody concentration after symptom onset	Days	3.59<br>(2.10, 5.94)	2.60<br>(1.62, 4.14)
**Baseline Antibody Concentration (y0)**	Initial antibody concentration before infection	ELISA Units	4.34<br>(1.98, 10.49)	4.64<br>(2.44, 10.44)
**Peak Antibody Concentration (y1)**	Maximum antibody concentration achieved at peak	ELISA Units	48.80<br>(25.61, 94.13)	232.55<br>(125.95, 453.51)

* HlyE = Hemolysin E, LPS = Lipopolysaccharide
