# Supplementary material for: Evaluating the accuracy of Salmonella Typhi Hemolysin E and lipopolysaccharide IgA to discriminate enteric fever from other febrile illnesses in South Asia
Source: medRxiv. 2025 Jun 22:2025.06.20.25329792. Preprint. [Version 1] doi: 10.1101/2025.06.20.25329792 (PMC12204246; doi:10.1101/2025.06.20.25329792)
Supplement: Supplement 6 [file media-6.pdf]

Appendix Table 5. Performance of Individual and Joint Antigen Cutpoints

Performance measure	Individual Antigen		Joint Antigen	
	HlyE IgA	LPS IgA	HlyE IgA	LPS IgA*
**Cutoff (EU)**	9.23	18.05	31.21	18.05
**Sensitivity**	0.76 (0.65, 0.85)	0.85 (0.78, 0.89)	0.88 (0.85, 0.92)	
**Specificity**	0.84 (0.71, 0.92)	0.90 (0.81, 0.96)	0.89 (0.84, 0.94)	
**Balanced Accuracy**	0.80	0.88	0.89	

* HlyE = Hemolysin E, LPS = Lipopolysaccharide
